# Effects of Tumor Necrosis Factor-*α* on Morphology and Mechanical Properties of HCT116 Human Colon Cancer Cells Investigated by Atomic Force Microscopy

**DOI:** 10.1155/2017/2027079

**Published:** 2017-06-21

**Authors:** Huiqing Liu, Nan Wang, Zhe Zhang, Hongda Wang, Jun Du, Jilin Tang

**Affiliations:** ^1^State Key Laboratory of Electroanalytical Chemistry, Changchun Institute of Applied Chemistry, Chinese Academy of Sciences, Changchun, China; ^2^University of Chinese Academy of Sciences, Beijing, China; ^3^Department of Microbial and Biochemical Pharmacy, School of Pharmaceutical Sciences, Sun Yat-sen University, Guangzhou, China

## Abstract

Chronic inflammation orchestrates the tumor microenvironment and is strongly associated with cancer. Tumor necrosis factor-*α* (TNF*α*) is involved in tumor invasion and metastasis by inducing epithelial to mesenchymal transition (EMT). This process is defined by the loss of epithelial characteristics and gain of mesenchymal traits. The mechanisms of TNF*α*-induced EMT in cancer cells have been well studied. However, mechanical properties have not yet been probed. In this work, atomic force microscopy (AFM) was applied to investigate the morphology and mechanical properties of EMT in HCT116 human colon cancer cells. A remarkable morphological change from cobblestone shape to spindle-like morphology was observed. In parallel, AFM images showed that the cellular cytoskeleton was rearranged from a cortical to a stress-fiber pattern. Moreover, cell stiffness measurements indicated that Young's modulus of cells gradually reduced from 1 to 3 days with TNF*α*-treatment, but it has an apparent increase after 4 days of treatment compared with that for 3 days. Additionally, Young's modulus of the cells treated with TNF*α* for 4 days is slightly larger than that for 1 or 2 days, but still less than that of the untreated cells. Our work contributes to a better understanding of colorectal cancer metastasis induced by inflammation.

## 1. Introduction

Inflammation is the physiologic response activated to repair the injured tissues and pathogenic agents. However, if inflammation becomes chronic, it can be harmful and may result in disease [1, 2]. A variety of clinical and epidemiologic studies have indicated that there is a strong link between chronic inflammation and cancer, and inflammation has been recognized as the “seventh hallmark of cancer” [3–7]. Chronic inflammation plays a critical role in tumor initiation, promotion, and progression by offering bioactive molecules from cells infiltrating the tumor microenvironment, such as chemokines, cytokines, and growth factors [1, 8]. Tumor necrosis factor-*α* (TNF*α*) is a key cytokine for building a complex link between inflammation and cancer [9, 10]. It was originally discovered as an antitumor cytokine [11]. But several lines of evidence now suggest that TNF*α* is one of the major mediators of cancer-related inflammation and acts as a crucial tumor-promoting factor. It mediates all steps of tumourigenesis, including cellular transformation, proliferation, invasion, angiogenesis, and metastasis and also accelerates tumor invasion and metastasis through induction of epithelial to mesenchymal transition (EMT) [10, 12].

EMT is essential for embryonic development, tissue remodeling, and wound repair [13, 14]. However, it is potentially destructive if deregulated. There is evidence that EMT plays an indispensable role in tumor progression, invasion, and metastasis [15–17]. During the process of EMT, cell-cell and cell-extracellular matrix (ECM) adhesions are changed with the loss of epithelial markers (such as E-cadherin) and the gain of mesenchymal markers (such as vimentin), leading to reorganization of the actin cytoskeleton and acquisition of the capability of moving and invading ECM [18–21].

In this paper, the main focus is on colorectal cancer (CRC), since it is a major health problem and is the fourth most common cause of cancer deaths worldwide [22]. Most deaths from CRC are ascribed to metastases and EMT is a highly relevant issue to CRC metastasis [23]. The mechanisms of TNF*α*-induced EMT in cancer cells have been well studied. However, mechanical properties (such as stiffness) have not yet been probed. Mechanical properties of the cells are fundamentally related to cell shape and motility and also considered as a biomarker for cellular cytoskeletal organization, which is the internal scaffolding composed of a complex network of three polymer biomolecules: actin microfilaments, intermediate filaments, and microtubules [24–27]. A powerful tool for studying the morphology and mechanical properties of the cells is the atomic force microscope (AFM), due to its outstanding spatial resolution and the high force sensitivity [28, 29]. Here, AFM was carried out to investigate the morphology and mechanical properties of EMT in HCT116 human colon cancer cells, which evaluated the effect of TNF*α* on cancer cells.

## 2. Materials and Methods

### 2.1. Cell Culture

HCT116 human colon cancer cells were obtained from the Type Culture Collection of the Chinese Academy of Sciences (Shanghai, China). HCT116 cells were cultured in McCoy'5A medium (Sigma, San Francisco, USA) containing 10% fetal bovine serum (FBS, Dingguo Company, Shanghai, China), 100 U/mL penicillin, and 100 *μ*g/mL streptomycin. All cells were incubated under a humidified atmosphere with 5% CO_2_ at 37°C and grew to a confluent monolayer for two or three days.

### 2.2. Cell Treatments

For AFM imaging and measurements, cells were split and seeded on 35 mm Petri dishes until reaching confluence. When the cells completely adhered to the bottom of the dish, they were left untreated or were treated with TNF*α* (MultiSciences Biotech Co., Ltd., Hangzhou, China) for 1, 2, 3, and 4 days in cell culture medium at a final concentration of 20 ng/ml. The medium was replaced using fresh TNF*α* every two days. Before the experiments, the cells were washed using phosphate buffer saline (PBS, 137 mM NaCl, 2.7 mM KCl, 8 mM Na_2_HPO_4_, 1.8 mM KH_2_PO_4_, pH 7.4) for 6–8 times.

### 2.3. AFM Imaging and Measurements

To accommodate the AFM imaging, the cells treated with or without TNF*α* were fixed by prewarmed 4% paraformaldehyde (PFA) for 15 min in PBS buffer at 37°C and then rinsed 10 times with PBS. All images were collected by NanoScope Multimode 8 (Digital Instruments, Veeco, USA). Topography images of fixed cells were recorded in contact mode with the silicon nitride AFM tips (DNP-10, 0.06 N/m, Bruker, USA) in PBS buffer. Simultaneously, the corresponding deflection images were also taken. The scan speed was set at 1-2 scan lines per second in the 512 × 512 pixel format.

A PicoSPM 5500 AFM (Agilent Technologies, Andover, USA) was carried out to measure Young's modulus of living cells in the cell culture medium. The cantilever (DNP-10, Bruker, USA) with nominal spring constant 0.06 N/m was used for the measurements. Before cell measurements the spring constant of the cantilever was calibrated on the cell-free bottom of the Petri dish by the thermal noise method [30] and found to be 0.072 N/m. For AFM measurements, approximately 1000 force-distance curves were collected on about 20 different cells. To probe the effect of TNF*α* on Young's modulus of living cells, the measurements were firstly performed on untreated cells with TNF*α*, then on the TNF*α* treated cells. The experiment was repeated four times for cells treated with or without TNF*α*.

## 3. Results and Discussion

### 3.1. TNF*α* Induces Morphologic Changes Consistent with EMT in HCT116 Cells

**Figure 1:**
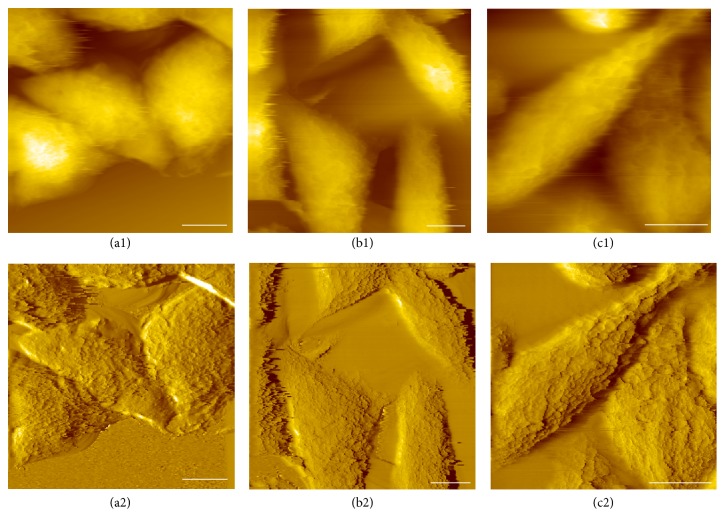
Morphological changes of HCT116 cells undergoing EMT. AFM height images of paraformaldehyde fixed cells treated without (a1) or with TNF*α* (20 ng/mL) for 2 days (b1) and 4 days (c1). Bottom panels (a2, b2, and c2) are the corresponding deflection images. Scale bar represents 15 *μ*m.

Metastasis induced by chronic inflammation is in charge of the majority of cancer-related deaths and also a major challenge during cancer therapy [31]. Clear evidence showed that chronically elevated TNF*α* in tissues could enhance the capacity of tumor cells to invade and metastasize [32, 33]. Here, morphological and cytoskeletal differences were firstly investigated by AFM to determine the effect of TNF*α* on HCT116 cells. The topography and deflection images of the cells fixed by PFA were obtained in contact mode (Figure 1). Deflection images are shown here due to their higher local contrast than topography images. As shown in Figures 1(a1) and 1(a2), the cells without TNF*α* treatment adhere to each other and exhibit a cobblestone-like phenotype. However, when the cells are treated with TNF*α* (20 ng/ml) for different time, there are changes in both morphology and intercellular space. After two days of treatment, the cells are slightly elongated and separation between adjacent cells is observed (Figures 1(b1) and 1(b2)). It was found that the average space between adjacent cells was about 4–10 *µ*m. After a four-day treatment with TNF*α*, the cells have transformed to a flattened and spindle-like morphology. Furthermore, intercellular separation is apparently increased and the intercellular space became greater than 12 *µ*m (Figures 1(c1) and 1(c2)). These changes are typical of cells with a mesenchymal phenotype.

**Figure 2:**
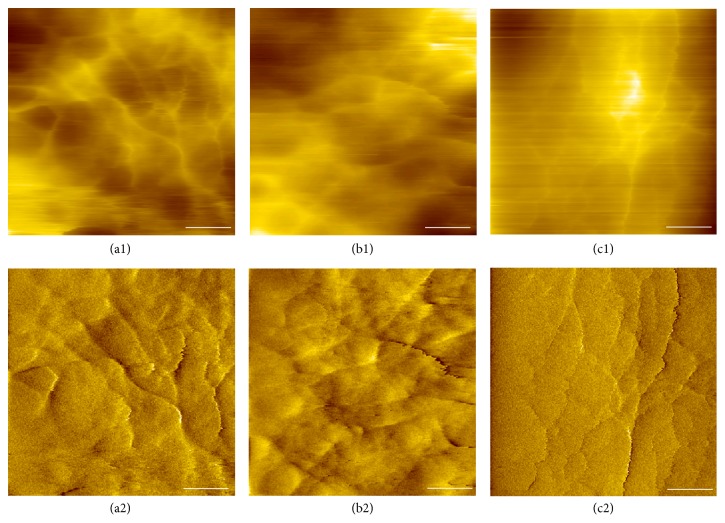
AFM topography images of HCT116 cells treated without (a1) or with TNF*α* (20 ng/mL) for 2 days (b1) and 4 days (c1), showing cytoskeletal organization under the cell membrane. Bottom panels (a2, b2, and c2) are the corresponding deflection images. Scale bar represents 1 *μ*m.

The shape of cells is strongly determined by the cellular cytoskeleton [34]. Therefore, the altered cytoskeleton in HCT116 cells can be expected after the cells were treated with TNF*α*. Owing to the higher resolution, AFM can be used to visualize the cytoskeletal organization of the cells [35]. The AFM images in Figure 2 illustrate the surface morphology of HCT116 cells treated with or without TNF*α*, which also directly indicate organization of the filamentous structures. As shown in Figures 2(a1) and 2(a2), the cytoskeleton of the untreated cell is organized in a meshwork of filaments, generating a stronger cytoskeletal structure. After two days of TNF*α* treatment, the original meshwork is disassembled. The cell comprises less well-defined filamentous structures, emerging as a randomly organized network with disrupted, short segments and leading to a weaker cytoskeletal structure (Figures 2(b1) and 2(b2)). After four days of treatment, the cell had an enhanced filamentous structure organized below its membrane compared with the cell treated for two days. The locally aligned filaments with tiny branch on their tops are observed, which is localized along the long axis of the adhered cell (Figures 2(c1) and 2(c2)). Presumably, the observed filamentous structures are actin filaments, because actin structure is the major component of cytoskeleton underneath the cellular membrane [36]. These results demonstrated that the gradual rearrangement of the cytoskeleton was obtained and induced by TNF*α* in time course. Collectively, morphologic and cytoskeletal changes of HCT116 cells were in agreement with an alteration to a mesenchymal-like phenotype after TNF*α*-induced EMT.

### 3.2. Changes in Stiffness of HCT116 Cells after TNF*α* Treatment

Mechanical properties of cells are primarily dependent on their cytoskeleton and also considered as a biomarker for cytoskeletal organization of cells [24, 37]. Thence, changes of cellular mechanical properties can reveal important information about alterations in their cytoskeleton, which, in return, further affects mechanical properties of cells [38]. To assess mechanical properties of HCT116 cells after being treated with TNF*α*, the values of Young's modulus were measured by force measurements for cells with or without TNF*α* treatment. To calculate Young's modulus from the force curves, Sneddon's modification of the Hertzian model for elastic indentation was used [39, 40]. The applied loading force *F* as a function of the indentation depth *δ* for a conical tip is described by the following:

(1) $$\begin{array}{c} F=\frac{2}{\pi }\frac{E}{\left(1-v^{2}\right)}\tan\left(\;\alpha \;\right)\delta^{2}, \end{array}$$

where *E* is Young's modulus, *α* is the half-opening angle of the tip (set to 25°), and* v* is the Poisson's ratio of the material, which is set to 0.5 for biological samples [41]. According to Hooke's law, the force *F* was obtained by multiplying the measured cantilever deflection *d*(*z*) by the spring constant *k*:

(2) $$\begin{array}{c} F=k\cdot d\left(\;z\;\right). \end{array}$$

On a hard material, the deflection of the cantilever *d*(*z*) will be equal to the piezodisplacement *z*; however, on a soft material, the deflection is reduced owing to elastic indentation:

(3) $$\begin{array}{c} d\left(\;z\;\right)=z-\delta . \end{array}$$

Based on the above equations, *E* is obtained as a function of measured quantities *z* and *d*(*z*). From fitting this function to the force-curve data, the values of Young's modulus were acquired. This calculation is discussed elsewhere in more detail [42, 43].

**Figure 3:**
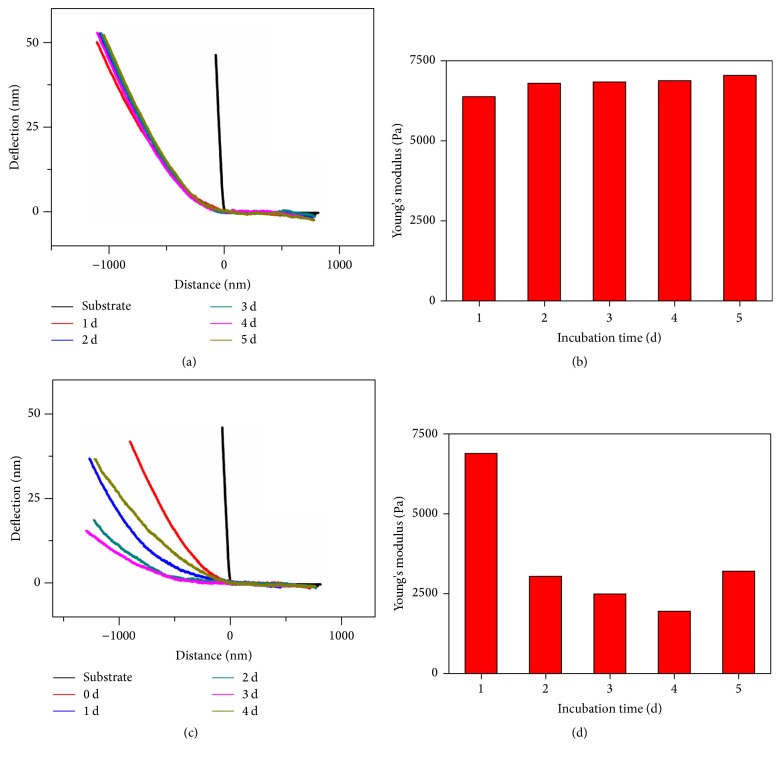
AFM measurements of the cell stiffness. (a and c) show the typical force curves measured on cells treated without (a) or with (c) TNF*α* (20 ng/mL) for the times indicated. (b and d) show the corresponding changes of the stiffness of cells without (b) or with (d) TNF*α* treatment.

Typical force curves measured on the surfaces of the living cells untreated or treated with TNF*α* for different time are plotted in Figures 3(a) and 3(c). Each curve represents different stiffness values. The measured curves were then analyzed with the Hertz model to determine the values of Young's modulus.  Figure 3 (panels on the right) shows the corresponding changes of Young's modulus for cells treated with or without TNF*α*. As for the untreated cells grown in the medium for different time, the force curves are highly overlapping (Figure 3(a)) and the measured Young's modulus values are practically consistent in the range of 6.3–7.1 kPa (Figure 3(b)). The results revealed that there were no clear changes in the cell stiffness during five days of culture without TNF*α*. When the HCT116 cells were treated with TNF*α* (20 ng/ml) for 1, 2, 3, and 4 days, the significant effect of TNF*α* on Young's modulus of living cells is apparent as demonstrated in Figures 3(c) and 3(d). Within 1 day, Young's modulus already reduced significantly from 6.9 kPa to 3.05 kPa. As time progressed, the cells experienced a larger reduction in stiffness compared with untreated cells. Young's modulus of the cells was 1.96 kPa after 3 days of treatment. However, the cells exhibited higher Young's modulus with TNF*α* treatment for approximately 4 days compared with the cells treated for 3 days, but still smaller than the untreated cells, indicating that the treated cells are less stiff and easier to deform than the untreated cells. These mechanical observations correspond well to the changes of the cellular cytoskeleton (Figure 2). Since the junctional complexes build adherens junctions and transduce mechanical forces through association with actin cytoskeletal networks [44], reduced adhesive interactions could also contribute to the decreased stiffness of the treated cells, which is supported by increased separation between adjacent cells. These findings reflected that the stiffness of HCT116 cells was decreased when they acquired a mesenchyme-like phenotype after 4 days of TNF*α* treatment. Additionally, during the process of EMT, there is an increased expression of mesenchymal-specific markers (i.e., *α*-smooth muscle actin (*α*-SMA) and fibronectin), which could determine the mechanical properties of a cell to some degree. It is tempting to hypothesize that the altered cytoskeletal organization and the increased expression of EMT-associated markers give the reasons for differences in the cytoskeleton and mechanics between cells treated with TNF*α* for different time. Based on the above observations, we can conclude that the observed alterations in mechanical properties were caused by the effect of TNF*α*. Quantitative measurement of HCT116 cell mechanical properties provides a novel window to assess cytoskeleton changes and cell predisposition and fate.

## 4. Conclusion

TNF*α*, a proinflammatory cytokine, is related to the wide spectrum of human diseases including cancer by accentuating EMT, which has been recognized as the first step of tumor invasion and metastasis. In this work, the morphological and mechanical differences of cells before and after treatment with TNF*α* (20 ng/mL) were investigated by AFM to evaluate the effect of TNF*α* on the HCT116 cells. The experimental results indicate that the cells lose connection to their neighbors and there is a change in morphology from cobblestone-like shape to spindle-cell-like shape when they are treated with TNF*α* for 4 days. Furthermore, Young's modulus for the treated cells gradually decreased from 1 to 3 days with TNF*α*-treatment, but it has an apparent increase after 4 days compared with that for 3 days. Additionally, Young's modulus of the cells treated with TNF*α* for 4 days is slightly larger than that for 1 or 2 days, but still less than that of the untreated cells. These changes in the mechanical properties are attributed to a rearrangement of cytoskeletal organization from a cortical to a stress-fiber pattern. Taken together, HCT116 cells underwent an EMT after being treated by TNF*α* for 4 days. These discoveries provide a better understanding for the role of TNF*α* in HCT116 cells metastasis and help quantitatively assess cell plasticity and fate.
